# Magnetic Harvester for an Autonomous Steel Health Monitoring System Based on Hall Effect Measurements

**DOI:** 10.3390/mi14010028

**Published:** 2022-12-23

**Authors:** Polychronis Pattakos, Spyridon Angelopoulos, Angelos Katsoulas, Aphrodite Ktena, Evangelos Hristoforou

**Affiliations:** 1 School of Electrical and Computer Engineering, National Technical University of Athens, 15780 Athens, Greece; chronispat@yahoo.gr (P.P.); spyrosag@central.ntua.gr (S.A.); angeloskatsoulas@gmail.com (A.K.); 2 General Department, National & Kapodistrian University of Athens, Evripos Campus, 34400 Evia, Greece; apktena@uoa.gr

**Keywords:** nondestructive testing, energy harvesting, Hall sensor, Internet of Things, steel health monitoring

## Abstract

In this article, the construction and experimental behavior of an Internet of Things (IoT)-compatible steel health monitoring system are examined. Falling under the general category of nondestructive testing, this new sensor is combined with an energy harvester to produce an autonomous automated device that can measure, store, and transmit measuring data without any need for human intervention. Based on common principles like the Hall effect, the monitoring system is put to use, and its results are presented.

## 1. Introduction

### 1.1. General

Nondestructive testing has been for decades the most efficient way of determining the quality of industrial materials such as steel. This umbrella term describes all the methods that are used on a raw material or component without altering its shape, composition, or condition and without interrupting its manufacturing, operation, or maintenance [1]. One of the ways of executing nondestructive testing, specifically for steel, is by examining its magnetic properties, which are inherent in all ferromagnetic materials [2].

In general, there are a number of different techniques used in the industrial sector to achieve this magnetic inspection [3]. Most of the sensors required for the required measurements are sensitive to fluctuations in magnetic flux density [4] or its related quantities like magnetic permeability [5] and Hall-effect-related voltage [6]. Similar applications include fluxgate sensors [7], SQUID sensors [8], and others.

### 1.2. Energy Harvesting

The ongoing development of micro-power electronic devices has led to a rise in demand for autonomous applications with alternative power sources, as chemical batteries are proving to be inefficient due to their size, limited durability, and the environmental concerns they raise. Because of its abundance and high energy density, vibration energy has been suggested as a viable solution and is categorized, according to each system’s operational principal, into the piezoelectric, electrostatic, and electromagnetic types [9].

The significance of energy harvesting becomes clear when considering mechanical systems, such as aircraft engines, where hardware monitoring during operation is mandatory, as any malfunction can prove catastrophic [10], or systems like wireless sensor networks (WSN), micro-electro-mechanical systems (MEMS), and unmanned aerial vehicles, which are designed to work specifically in severe environments [11] and battery replacements are not an option.

Another widely utilized harvesting principle is one where the magnetostrictive properties of materials are used to obtain voltage and therefore electrical power. However, when using these kinds of harvesters, it is difficult to match their own resonant frequency with the ambient vibration of the environment, and, therefore, the harvested power will often be below the mW level [12].

### 1.3. Hall Effect

One of the most basic concepts related to the study of the electromagnetic field is the Lorentz force, which is defined as the vector sum of the force exerted on a charge moving at velocity ***v*** inside a magnetic field ***B*** and the force exerted by the electric field ***E*** on the same charge *q* [13]. This is shown in Equation (1):

***F*** = *q*·***E*** + *q*·(***v*** × ***B***) (1)

the Lorentz force couples electromagnetics to mechanical phenomena and has found a lot of applications in sensors and actuators. It is also related to magnetotransport material properties, like the Hall effect [13].

The Hall effect is based on the transverse Lorentz force experienced by charge carriers inside a magnetic field, ***B***. When a current-carrying conductor is placed inside a magnetic field, it experiences a Lorentz force perpendicular to the plane defined by the field and the current.

### 1.4. Magnetic Circuits

**Figure 1:**
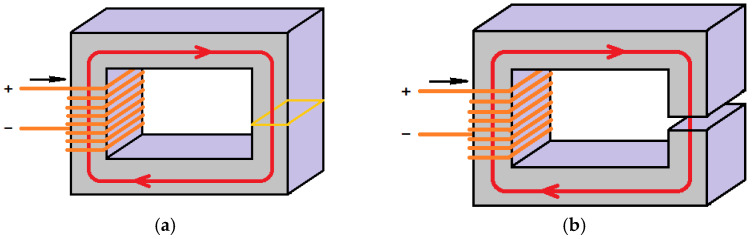
Examples of magnetic circuits (**a**) without magnetic resistance; (**b**) with an air gap acting as magnetic resistance.

A circuit can be defined as a closed path in which the flow of some quantity takes place. In electrical circuits, electrical current flows through conductors and other—passive or active—devices comprising it. The purpose of magnetic circuits is to guide the flux Φ along a desired route in order to generate a magnetic field of a particular spatial profile, “sense” an existing magnetic field, or link in a contactless way two circuits or components (Figure 1) [6].

As will be discussed below, the Hall sensor and the under-test sample form a magnetic circuit in which the magnetic flux is provided by a permanent magnet.

### 1.5. Design Requirements

The Internet of Things [14] has already been adopted in many industrial applications. Hall effect sensors have been commonly used for years, based on the principles provided by magnetic circuit analysis for nondestructive testing. However, these measuring systems and methods are now redesigned to meet new requirements like energy efficiency, data collection, and data transmission, as previously described in [15]. In this paper, an effort is made to combine an energy harvesting system with a new steel health monitoring device based on Hall sensors designed for autonomous operation. The purpose of such a device, once implemented, would be to monitor the condition and mechanical stress that various components are subjected to during their operation. An example of its use in real-world conditions would be for the live monitoring of a ship’s hull or machinery that are sensitive to stresses during operation at sea.

## 2. Materials and Methods

### 2.1. Energy Harvester System

#### 2.1.1. System Description

**Figure 2:**
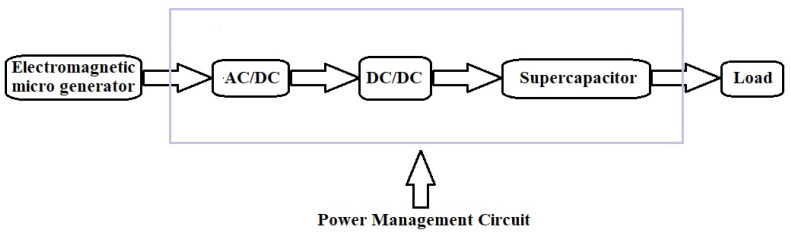
Harvester system architecture.

The energy harvester system can be broken down into two main parts, which are the energy generator and the energy storage circuit. Connecting the two parts is a power management circuit. The system’s general architecture is presented in Figure 2.

#### 2.1.2. Electromagnetic Microgenerator

**Figure 3:**
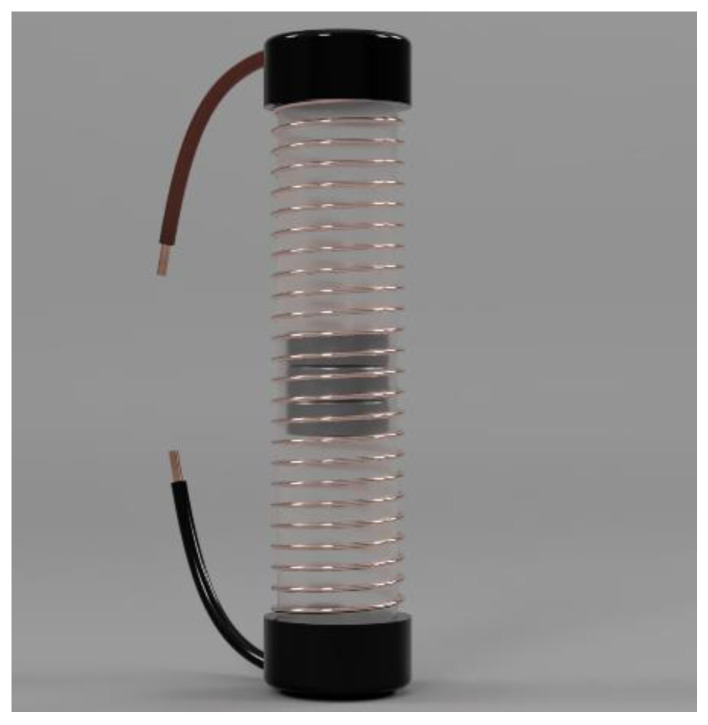
Microgenerator with inertial mass inside the coil (3-D rendering).

The electromagnetic microgenerator is a system that transforms the vibrations of an external system into storable energy. This is constructed by placing a set of permanent magnets inside a tube that has a coil wrapped around it. The magnets are the inertial mass that is needed to levitate inside the tube. Their movement is enhanced by placing another two magnets of opposite polarity on the sides of the tube (Figure 3).

When an external vibration is applied, the magnets begin to oscillate. The oscillation frequency depends on the environment in which the harvester is placed. A typical application includes the exploitation of vibrations that occur in a ship. This particular harvester was designed for this application. The oscillations’ result is the creation of a differential magnetic field inside the coil, which, in turn, creates an alternating voltage at the coil’s terminals [16].

**Figure 4:**
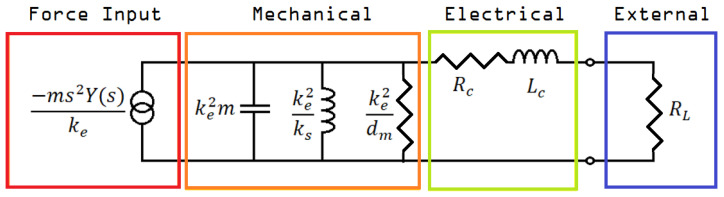
Circuit equivalent of an electromagnetic microgenerator (force input—mechanical parts) connected to an electromagnetic transducer (electrical—external parts).

In [16], the electromagnetic microgenerator, when connected to an electromagnetic transducer, is described by an electrical circuit equivalent as shown in Figure 4.

In this circuit, *m*, *s,* and *Y(s)* represent the inertial mass, the vibration whose energy is to be harvested, and the oscillation that is caused by the external force; *k_e_*, *k_s_*, and *d_m_* are the system’s convection factor, elastic properties factor, and mechanical damping; *R_c_*, *L_c_*, and *R_L_* are the internal resistance and inductance of the generator, and the internal resistance of the connected load, respectively. This circuit equivalent can be found if we consider the relative motion *z(t)* between the motion of the mass *x(t)* and the displacement of the case of the generator *y(t)*. The equation of motion for the inertial mass would be:

(2) $$m\cdot \ddot{z}\left(t\right)+d\cdot \dot{z}\left(t\right)+k_{s}\left(t\right)=-m\cdot \ddot{y}\left(t\right)$$

(3) where z(t)= x(t)−y(t)

then by rearranging Equation (2) and considering the Laplace transform, we get:

(4) $$-m\cdot s^{2}\cdot Y\left(s\right)=s\cdot Z\left(s\right)\cdot \left[m\cdot s+d+\frac{k_{s}}{s}\right]\;$$

which can be viewed as the mechanical analogue of the Norton equivalent electrical circuit described by the equation:

(5) $$-I_{1}\left(s\right)=E\left(s\right)\cdot \left[s\cdot C+\frac{1}{R}+\frac{1}{s\cdot L}\right]\;$$

when electrical and mechanical parameters have been replaced accordingly.

For a sinusoidal input, $\;y\left(t\right)=Y_{0}\cdot cos\left(\omega t\right)$ , at resonant frequency $\omega_{n}$ , ( $\omega_{n}{\text{​}}^{2}=k_{s}/m$ ) the power delivered to the load, $p_{L}$ , is calculated to be:

(6) $$p_{L}\left(\omega_{n}\right)=\frac{k_{e}{\text{​}}^{2}m^{2}Y_{0}{\text{​}}^{2}\omega_{n}{\text{​}}^{4}R_{L}}{d_{m}{\text{​}}^{2}\left[{\left(R_{C}+R_{L}+\frac{k_{e}{\text{​}}^{2}}{d_{m}}\right)}^{2}+{\left(\omega_{n}L_{C}\right)}^{2}\right]}.\;$$

as a result, some basic parameters of the circuit are summarized in the following list [17]:

1. The inertial mass inside the coil must be the largest possible;
2. The external oscillation’s frequency and the resonant frequency of the inertial mass must be as close to equal as possible;
3. The oscillation of the inertial mass must not be prohibited by the shape of the generator;
4. *R_c_* (the generator’s internal resistance) must be one or more orders of magnitude less than *R_L_* (the resistance of the load);
5. The system’s mechanical damping should be as little as possible;
6. *k_e_* (the electromagnetic constant) should be such that the generator’s mechanical internal resistance is close to equal to the load’s resistance (*R_L_* ≈ *k_e_*^2^/*d_m_*).

The design of the generator was completed by adhering to the following steps:

1. A plastic cylinder is chosen to act as a housing tube;
2. As an inertial mass, permanent neodymium magnets (NdFeB) were chosen because of their high density and strong magnetic field;
3. Permanent neodymium magnets were also used, one on each side of the tube, to enhance the inertial mass’s levitation;
4. As a final step, the coil, whose length and number of turns would be determined through trial and error, is wound around the shell.

**Figure 5:**
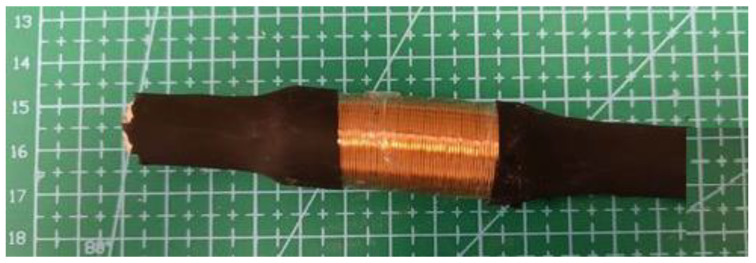
A finished microgenerator.

The finished microgenerator is shown in Figure 5.

After comparing two sets of microgenerators, one with Ø10 mm and one with Ø8 mm magnets, two generators were deemed to be the most efficient. Both of these generators were 50 mm in length, had 600 turns, and contained a set of 20 magnets. Then, 3 such generators were combined, creating a larger microgenerator system of two Ø10 mm and one Ø8 mm magnets.

**Figure 6:**
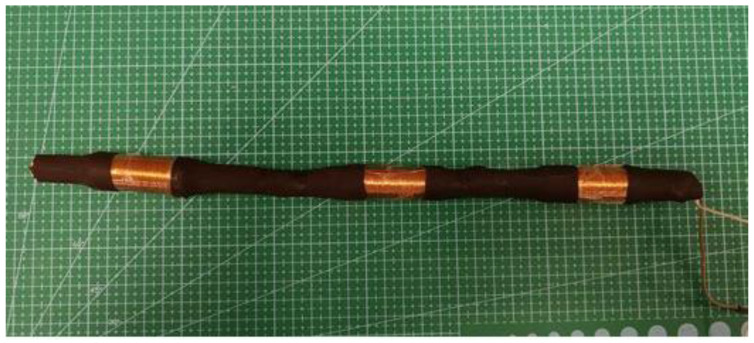
Microgenerator system of three generators.

The three generator system is shown in Figure 6.

**Figure 7:**
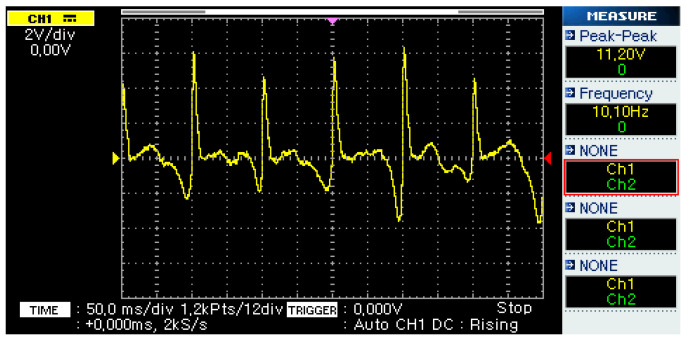
Voltage output of the microgenerator.

**Figure 8:**
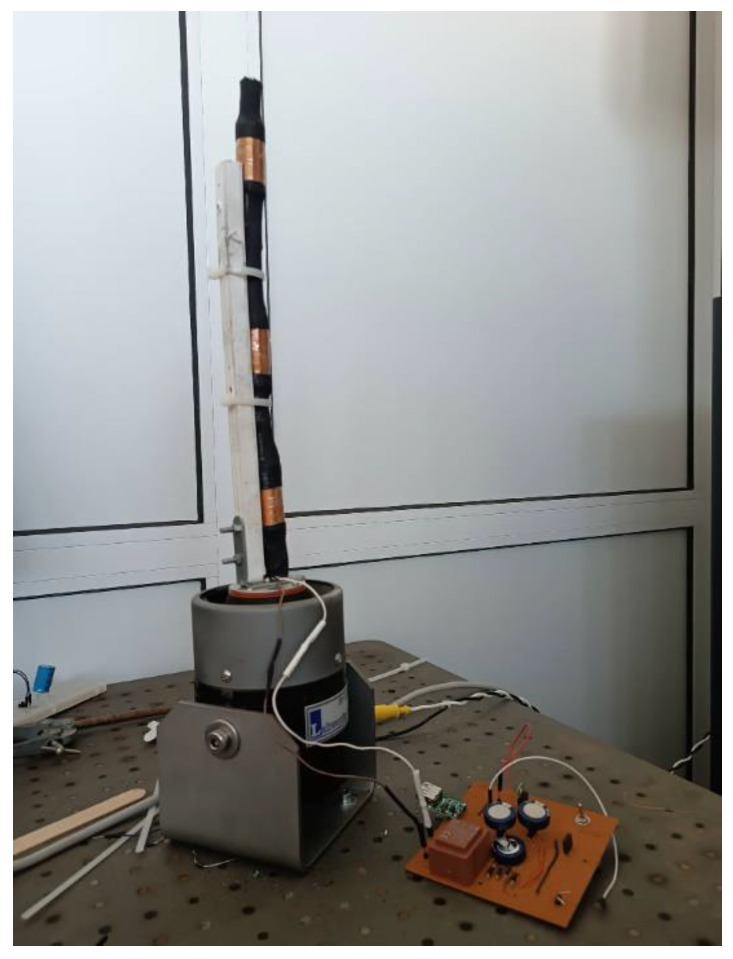
Oscillation generator connected to the power management circuit.

Figure 7 shows the output of the microgenerator system when an external vibration is applied. The external vibration had a frequency of 10 Hz, which is a common vibration frequency found in ships [15]. This output was achieved during the experiment described later in this paper by attaching the energy harvester to a laboratory oscillation generator (see Figure 8), excited by a sinusoidal signal, resulting in an acceleration of 10 m/s^2^ peak-to-peak.

#### 2.1.3. Power Management and Energy Storage Circuit

The microgenerator’s energy output must be converted and stored in order to be used by the measuring device. The conversion stage is based on a full-wave bridge rectifier, while the storage stage is based on the use of supercapacitors. As shown in Figure 7, the output of the microgenerator is an alternating voltage. Thus, the bridge rectifier circuit converts it to direct voltage. The bridge rectifier is made up of four diodes, which can have a significant voltage drop compared to the harvester’s output, resulting in a low voltage supply to the energy-storing supercapacitors. This effect is minimized by using a transformer to increase the generator’s output to a higher voltage, along with four Schottky diodes with a nominal voltage drop of 150 mV.

**Figure 9:**
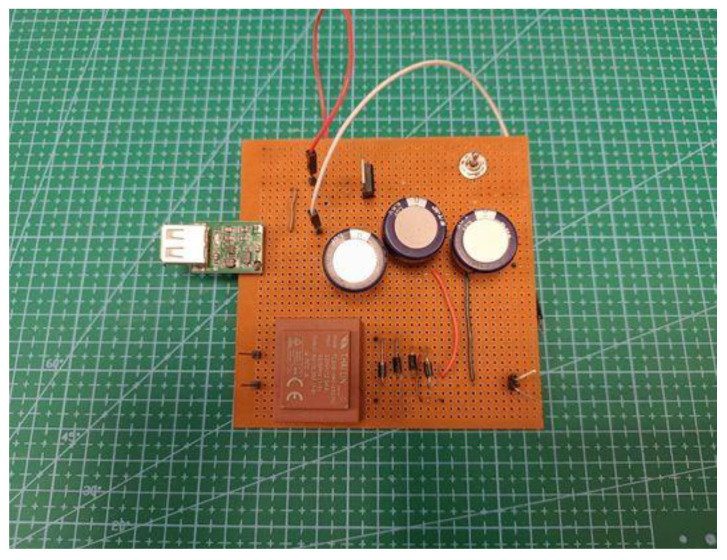
Power management, storage, and output circuits prototype.

The system prototype is shown in Figure 9. Energy storage is achieved using three supercapacitors connected in series in order to further increase the total voltage. Their nominal capacitance was equal to 1 F, and their maximum voltage was 5.5 V. As a result, the combined equivalent capacitor would have a capacitance of 0.33 F and a voltage of 16.5 V. The nominal energy storage capacity is calculated in Equation (2):

(7) $$E=\frac{1}{2}\;\cdot \;C\cdot V^{2}=45.3\;J$$

### 2.2. Hall Sensor

#### 2.2.1. Sensing Yoke

**Figure 10:**
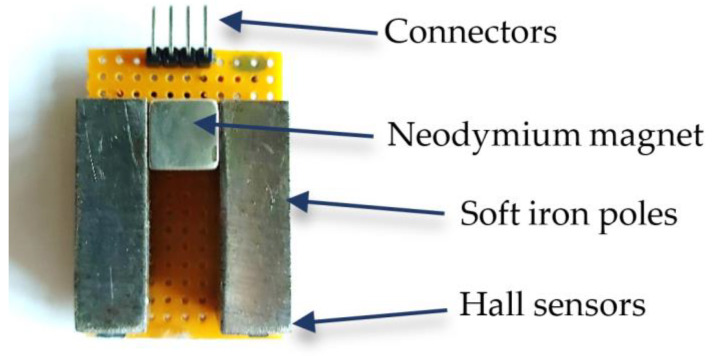
Sensing arrangement consisting of a cubic permanent magnet, two ferromagnetic poles, and two Hall sensors.

The developed sensing arrangement consists of two ferromagnetic poles and a cubic neodymium permanent magnet, forming a yoke, as shown in Figure 10. At the free end of each pole, a Hall sensor is placed. A detailed description of the operating principle of the arrangement can be found in [17]. However, the dimensions, the electronics, as well as the packaging of the current arrangement have been modified in order to produce a more sensitive, robust, and energy-efficient device.

The two poles are made of soft iron, having a length of 30.5 mm and a width of 9.5 mm. Between the two poles, a cubic 9.5 mm NdFeB permanent magnet was placed. Finally, a linear bipolar SS49E Hall sensor (Honeywell, Charlotte, NC, USA) was placed under the free end of each pole. The arrangement was mounted on a board, which had the appropriate terminals for the connection of the two Hall sensors (output of Hall sensor 1, output of Hall sensor 2, supply voltage, and ground).

#### 2.2.2. Electronics

**Figure 11:**
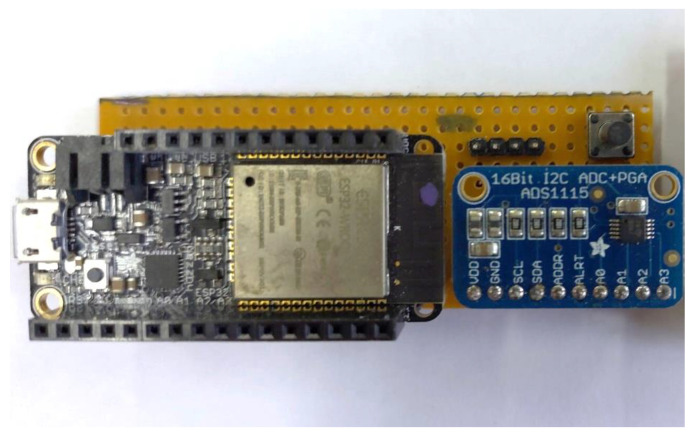
The electronics board, including the microcontroller’s board and the external ADC.

Figure 11 shows the electronics board that was developed. The development board that was used includes an ESP32 microcontroller (Espressif Systems, Shanghai, China) with a processor operating at 240 MHz, a 12 bit Analog-to-Digital Converter (ADC), and integrated USB, Wi-Fi, and Bluetooth connectivity. As a result, the device can operate either wired or wirelessly, supplied by a standard USB port or a lithium battery. Moreover, the ESP32 microcontroller includes deep sleep modes, enabling its low-power operation.

However, the 12 bit resolution of the embedded ADC was found to be insufficient for the specific application. As a result, an external 16 bit ADC (ADS1115, Texas Instruments, Dallas, TX, USA) was added. This is also a low-power device that offers 4 analog inputs with adjustable gain and communicates via the I^2^C protocol.

**Figure 12:**
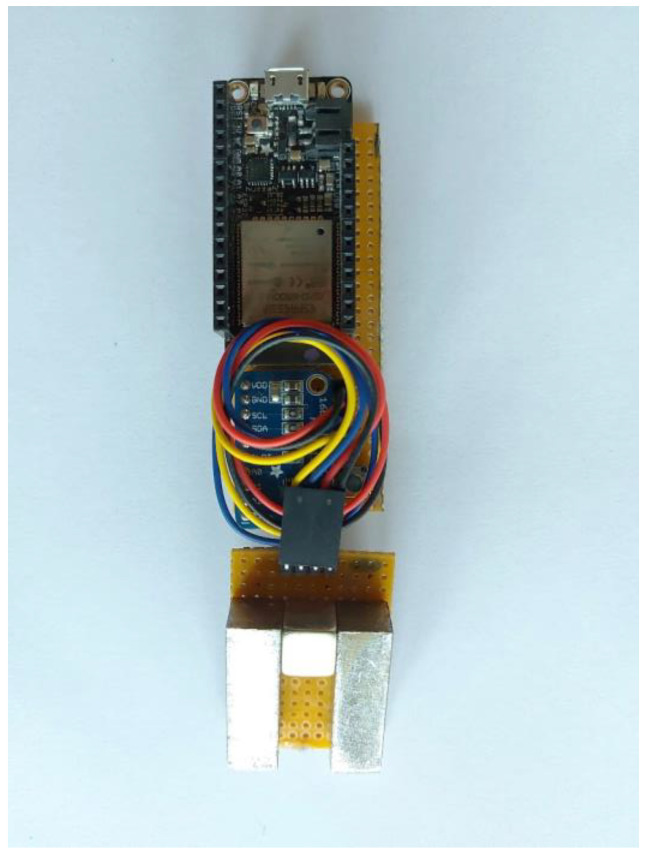
Complete arrangement, including both the electronics and the sensing yoke.

The complete arrangement, including both the electronics board and the sensing yoke, is shown in Figure 12.

#### 2.2.3. Packaging

**Figure 13:**
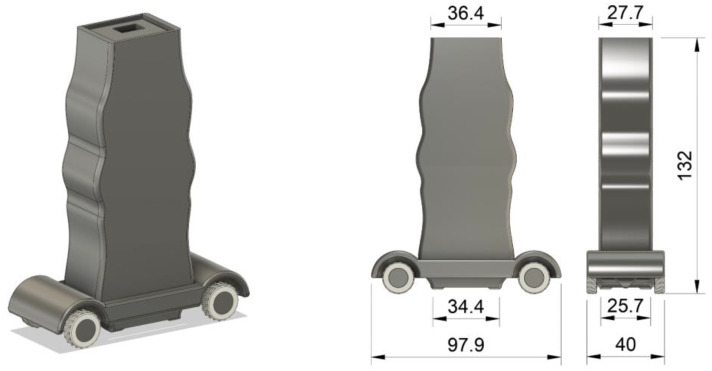
Three-dimensionally printed enclosure of the device (dimensions in mm).

In order to use the arrangement as a robust standalone device, a 3D-printed enclosure was designed and manufactured. Its design and dimensions are shown in Figure 13. The arrangement can be inserted into the case through its sliding top lid. Afterwards, it can be operated either wired, using a standard USB cable, or wirelessly, via Wi-Fi or Bluetooth. Furthermore, the design includes a detachable base with rubber wheels, which enables the friction-reduced movement of the device on any surface, provides the option to set the desired lift-off, and prevents the device’s wear due to friction.

**Figure 14:**
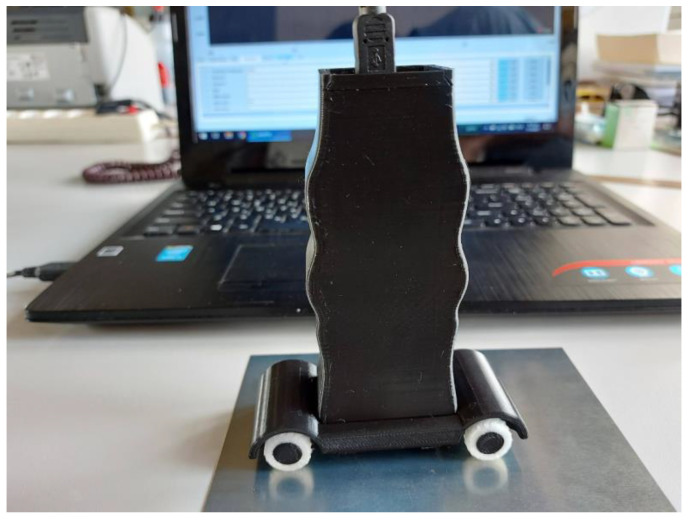
The final device in its 3D-printed packaging, connected to a laptop via a USB cable.

The final device is shown in Figure 14. The enclosure and the base were 3D-printed using PLA (polylactic acid) material, while the wheel tires were made from TPU (thermoplastic polyurethane) material.

### 2.3. Completed System Overview

**Figure 15:**
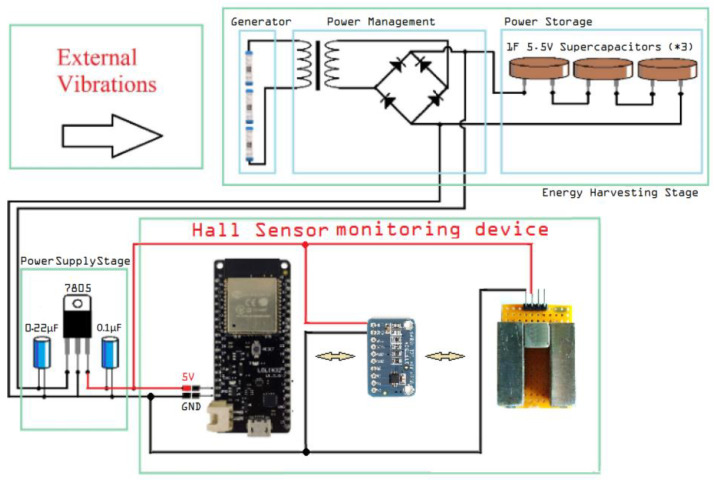
Autonomous Hall-effect sensor system overview.

In Figure 15, an overview of the completed autonomous Hall effect sensor system is presented. The nominal operating currents of the ESP32 are 10 μA during deep sleep and 260 mA during normal operation at 3.7 V [18]. For a full cycle of 10 min with only 0.5 s of operation, it can be calculated that it consumes less than 0.5 J. During its 0.5 s operation, the energy consumption is close to 0.16 J [15].

Because of the very short period of operation, the ADS1115 converter [19] and the two Hall sensors have an insignificant energy consumption. Such small consumption (nominally less than 0.2 J) results in a total consumption of less than 0.4 J for a 10 min operating cycle. For a full charging time of 20 min, which results in a nominal amount of energy stored equal to 45.3 J, it is safe to say that the system can achieve autonomous operation.

## 3. Results

### 3.1. General Description

**Figure 16:**
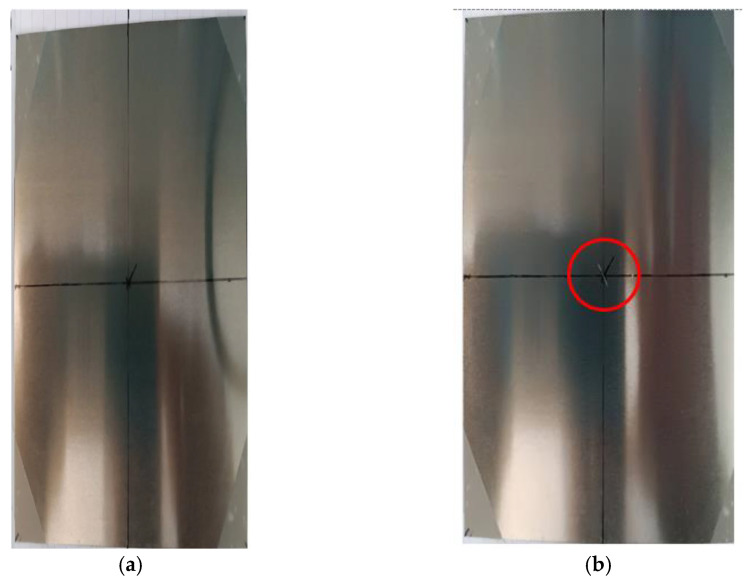
Image of the sample: (**a**) before the flaw; (**b**) with a flaw at its center.

In order to test the behavior of the sensor, an experiment was designed where the same electrical steel sample was tested before and after the creation of a small surface scratch. The scratch was made on the sample’s center to avoid uncertainties related to the magnetization at the edges of the sample. The sample, before and after the scratch was added, can be seen in Figure 16.

**Figure 17:**
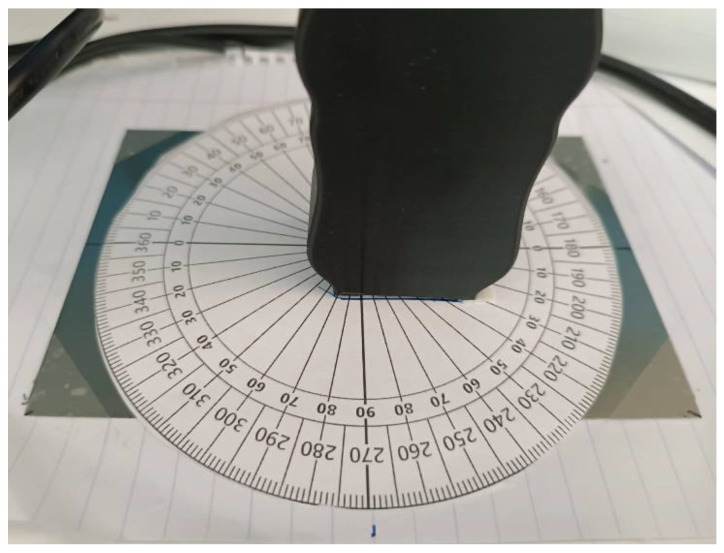
Experimental configuration.

The monitoring device was placed on the sample, with one of its two Hall sensors placed directly above the center. A paper protractor was also attached to the device for reference, as seen in Figure 17.

By measuring the magnetic flux emanating from the sample at different directions, the magnetic anisotropy profile of the under-test material may be obtained [20]. The term anisotropy is used to explain any preference a material’s property may exhibit, macroscopically, for a given direction or directions. In the case of magnetic materials, anisotropy reflects the tendency of the magnetization vector to lie along one specific direction [6]. In this particular case, we wanted to test whether or not there was any correlation between the angle of the monitoring device to the sample and the magnetic field measured by the Hall sensor. Measurements were taken every 5 min, each time turning the monitoring device manually by 5 degrees. The axis of rotation was perpendicular to the plane of the sample and passed through its center, as shown in Figure 16. The 5 min were enough to recharge the capacitors of the energy harvester, as predicted. During the measurements, external vibrations were provided by the oscillation generator shown in Figure 8.

### 3.2. Measurements

**Figure 18:**
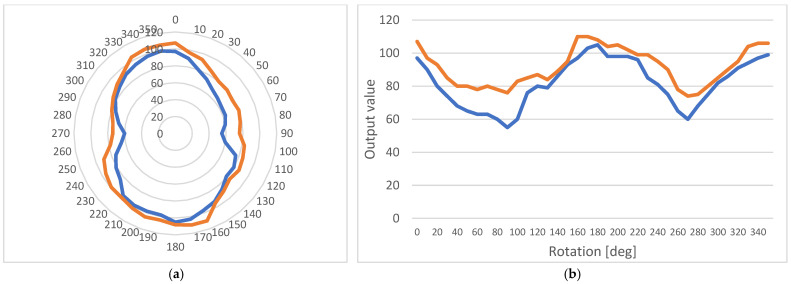
Anisotropic behavior of the sample before (blue line) and after (orange line) the flaw was added: (**a**) A polar diagram; (**b**) A linear diagram.

Figure 18 shows the measurements performed on the sample before and after the flaw was added. It is clear that the anisotropic behavior can still be detected, although there is a change both in its peaks and its range.

### 3.3. Additional Offset Measurements

**Figure 19:**
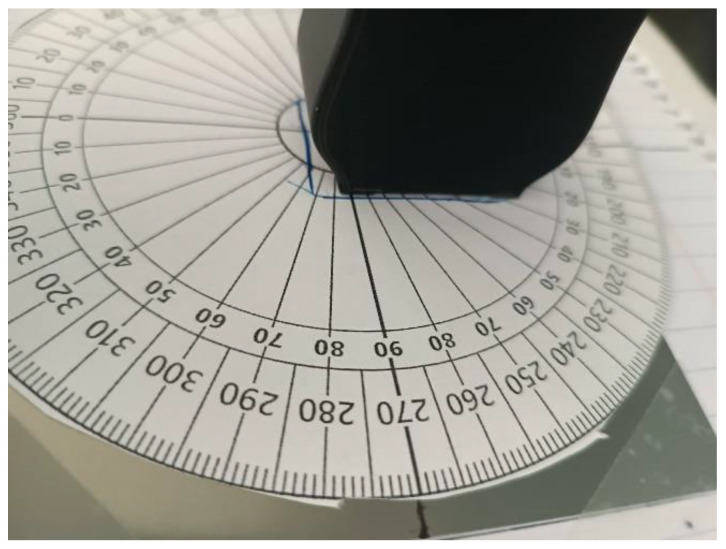
Experimental configuration offset by 5 mm; previous position marked by a blue outline.

For reasons that will become apparent in the Discussion Section of this article, another set of measurements was performed, this time by offsetting the rotation axis of the monitoring device by 5 mm, as shown in Figure 19.

**Figure 20:**
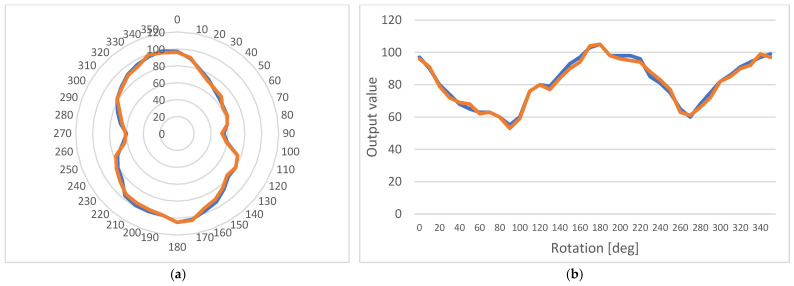
Anisotropic behavior of the sample before the flaw; measurement on point (orange line) and with a 5 mm offset (blue line): (**a**) A polar diagram; (**b**) A linear diagram.

**Figure 21:**
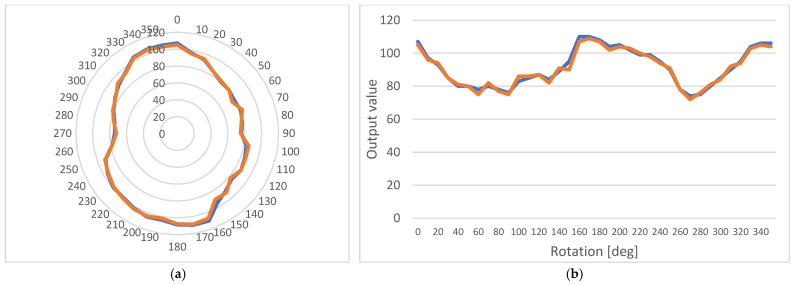
Anisotropic behavior of the sample after the flaw was added; measurement on point (orange line) and with a 5 mm offset (blue line): (**a**) A polar diagram; (**b**) A linear diagram.

This offset resulted in a set of measurements being taken around the point of examination instead of directly above it. The two sets of measurements, before and after the flaw, are shown in Figure 20 and Figure 21, respectively.

The above figures indicate that, when the two sets are compared, flaw influence on results is greater than the influence of position change.

## 4. Discussion

**Figure 22:**
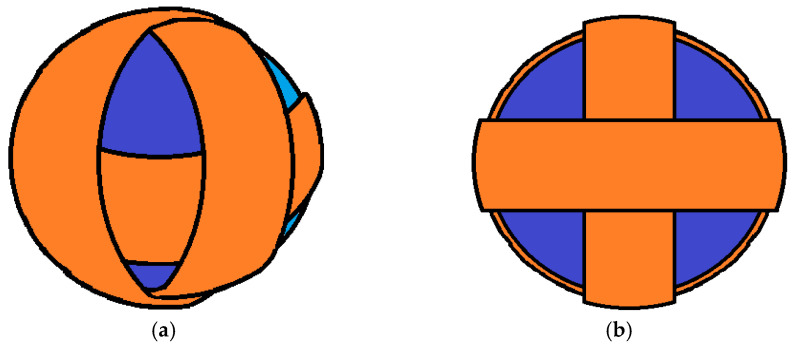
Three-dimensional design of future work configuration for a three-dimensional harvester: (**a**) Side view; (**b**) Front view.

Examination of the harvester’s operation shows that although it meets the energy demands of this application, its sufficiency could be improved by rethinking its design. This is because (a) the inertial mass inside the microgenerator is subject to internal friction that is the cause for energy loss, and (b) external settings like the naval environment mentioned above usually have oscillations in more than one dimension, which is kinetic energy that could also be harvested. To account for both of these reasons, a spherical design is proposed, as seen in Figure 22.

As a result, a new design is proposed for a future work, where the harvester’s cylindrical tube is replaced with a sphere with three coils wrapped around it to account for all three spatial dimensions. Acting as inertial mass, a set of permanent magnets, similar to the original generator, can be used. However, instead of levitating, they would be coated in rubber that would allow bouncing of the sphere’s internal walls through elastic collision.

Expanding on this design, since the frequency of the external vibration would typically be different in each direction of 3-dimensional space, the spherical shape could be replaced with that of an ellipsoid. The length of the ellipsoid in each direction would have to be determined as future work through theoretical analysis and experimentally to achieve the maximum efficiency, accounting for the size of the inertial mass, the length of each coil, and the external frequency of the oscillation.

By examining the measurements of the experiment, there are three results that would indicate the direction that future work should proceed in:

- The autonomous operation of the device is safely achieved due to the low power consumption of the chosen components;
- The sensor can easily detect the material’s anisotropic behavior, which is affected by flaws in its composition;
- For the purpose mentioned above, the proposed design gives us the possibility to perform measurements close to, but not directly above, the examination point.

**Figure 23:**
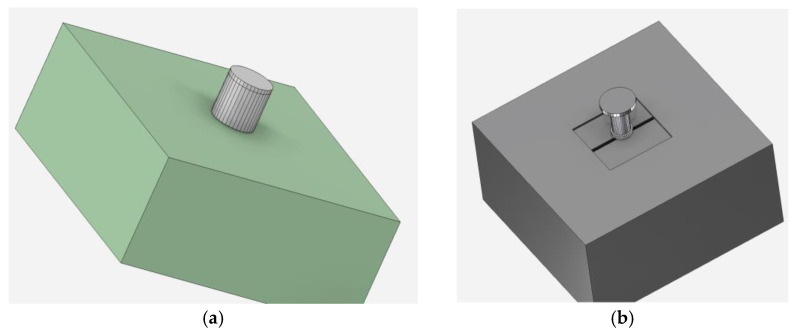
Three dimensional imaging of future work configuration for the sensing element (created with ANSYS R19.2 software, licensed to NTUA): (**a**) Element over a flawless part; (**b**) Element over a part with a simulated flaw.

These three points suggest that one of the magnetic properties of each point that should be examined is its anisotropy. So, a new configuration for the sensing element of the monitoring device is put forward for any future work. This new test configuration is shown in Figure 23.

**Figure 24:**
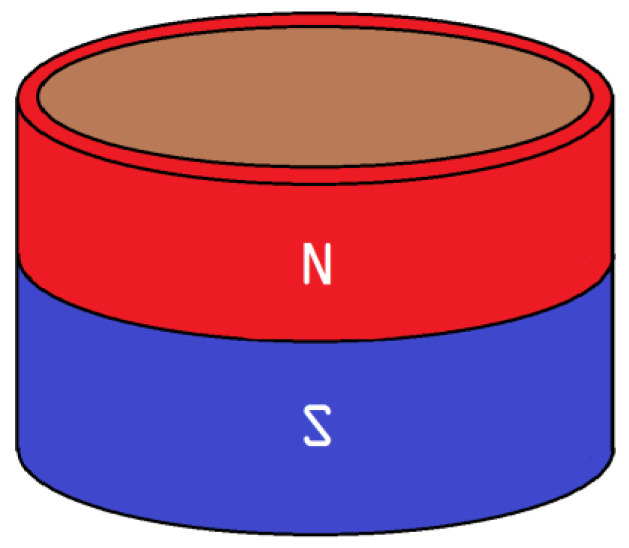
Shape of a permanent magnet.

In this configuration, a set of multiple correctly calibrated Hall sensors is placed in a circle between the central cylinder and the measuring point. The outer hollow cylinder that is missing in the second image (Figure 23b) is a permanent magnet with its poles directed as shown in Figure 24. The sensors are placed in the part resembling a toroid in Figure 23b. Their exact number could vary according to the specifications of the experiment, such as energy consumption and dimensions.

**Figure 25:**
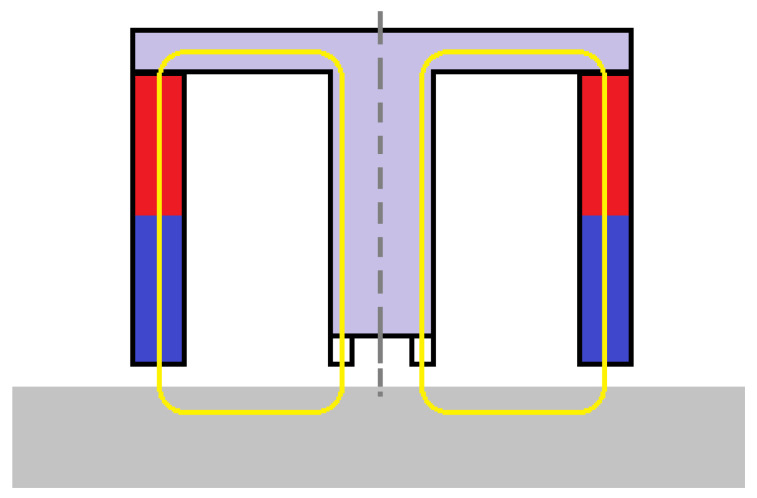
New configuration cross-section with two magnetic circuits.

Figure 25 shows that any cross section of this configuration would simulate two magnetic circuits similar to the ones in the monitoring device in the experiment.

This would give us the benefit of performing a number of measurements simultaneously, using all the sensors at once, without having to rotate the monitoring device to perform one measurement at a time as in our previous experiment. Since the offset measurements show similar results to those taken directly above the point, it is easily concluded that this configuration could provide enough accurate data for the sample’s condition. Furthermore, a number of these devices could be connected in an array type configuration so that data could be exchanged and transmitted in an Internet of Things-compatible arrangement.

## 5. Conclusions

Nondestructive testing is an ever-expanding field in engineering, especially today with the Internet of Things providing new standards for efficiency, power consumption, and data processing. As we have seen in the process of this experiment, there is always opportunity for developing new methods and devices for acquiring these data, which make for improved industrial applications. Additionally, the principles of autonomous operation for sensors are a game-changing concept for nondestructive testing, as they can provide synchronous data even for the most specific geometries and physical qualities of test subjects.

## Data Availability

Not applicable.
